# Bilateral Total Knee Replacement in a Patient With Poliomyelitis: A Case Report From Eastern Morocco

**DOI:** 10.7759/cureus.33317

**Published:** 2023-01-03

**Authors:** Saber Zari, Otmane Sammouni, Najib Abdeljaouad, Hicham Yacoubi

**Affiliations:** 1 Department of Traumatology and Orthopedics, Mohammed VI University Hospital, Oujda, MAR; 2 Faculty of Medicine and Pharmacy, Mohammed First University, Oujda, MAR

**Keywords:** functional outcomes, instability, total knee arthroplasty, genu recurvatum, poliomyelitis

## Abstract

Total knee replacement in limbs affected by poliomyelitis can be complicated by anatomical features, narrowing of the intramedullary canal, quadriceps muscle weakness, flexion contractures, and ligament laxity. Total knee arthroplasty (TKA) leads to good results in returning to daily activities and overall functional improvement of these polio patients by restoring near-normal joint mobility, pain relief despite impaired quadriceps strength, and bone and soft tissue defects. Our case report is about a patient with sequelae of bilateral poliomyelitis of the limb benefiting from a hinge-type total knee prosthesis. The rotating hinge total knee prosthesis has certainly been a revolution in the surgical treatment of patients with poliomyelitis, bringing considerable functional improvement. Nevertheless, total knee replacement on poliomyelitis limbs is still a therapeutic challenge, even for the most experienced hands.

## Introduction

It is known that poliomyelitis is a virus infection that typically affects children but can also affect adults and is very contagious. The virus spreads mainly through the fecal-oral pathway, targeting the nervous system and causing paralysis in one week. Less than 1% of those affected develop paralysis, which typically targets the lower extremities [1]. Widespread use of the poliovirus vaccine has reduced the incidence of polio in many Western countries. Although polio has been eradicated, 40,000 to 55,000 people, with an average age of 55 to 60 years, still suffer from polio-related illnesses [2]. According to the literature, there are limited cases of total knee arthroplasty (TKA) in poliomyelitis-affected extremities [3,4,5]. Despite the fact that this kind of surgery has generally been successful in reducing pain, there have been worries regarding recurrent knee instability [3,4,6]. Here, we describe the successful bilateral TKA surgery of a 56-year-old woman with a history of polio infection.

## Case presentation

**Figure 1:**
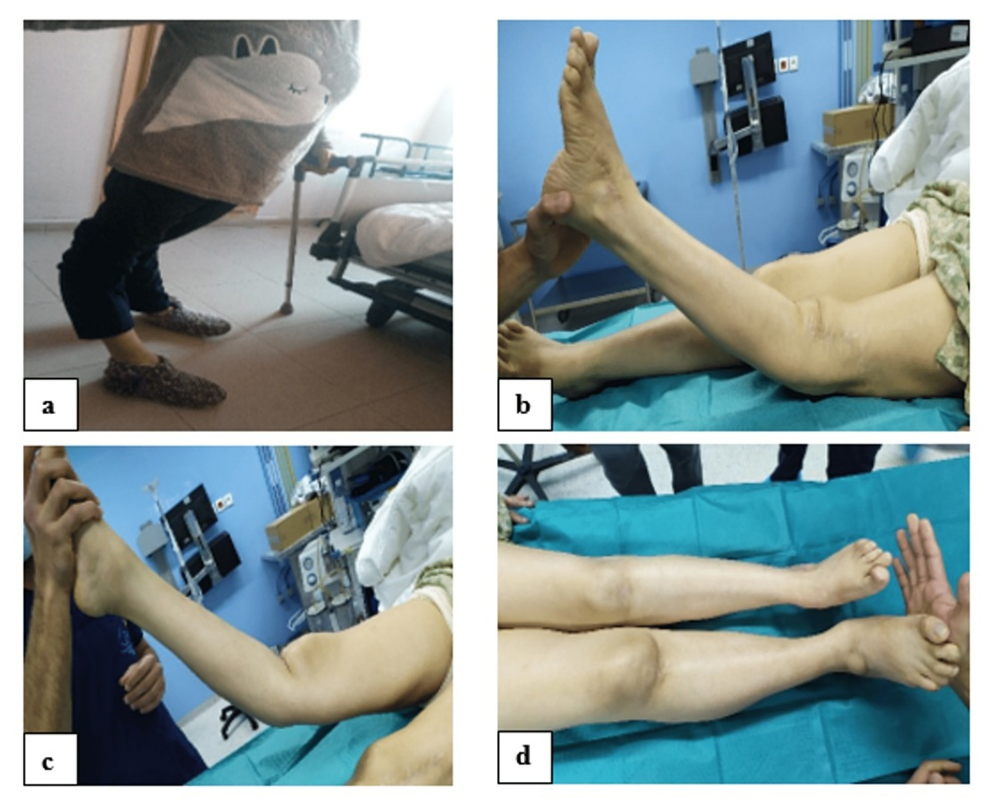
Clinical images showing a) knee recurvatum compensated by extension of the hip and dorsiflexion of the foot. b) Significant recurvatum in the supine position of the left knee, c) recurvatum of the right knee, and d) 3 cm shortening.

A 56-year-old female had bilateral chronic knee pain. She was diagnosed with poliomyelitis in childhood, causing her spinal paralysis. At the age of seven, the patient was walking with a cane and had no trauma history. Currently, the patient presented with progressive pain and limited range in both knees. On physical examination, the patient has bilateral valgus and bilateral knee recurvatum of 40°, a shortening of 3 cm of the left lower limb (Figure 1). The quadriceps force of both knees was 3/5. The left knee range of motion (ROM) was 40-0º-60º, while the right knee ROM was 40º-0-80º. Both knees had global laxity (anteroposterior and mediolateral), with more severe on the left side. The patient has a valgus flat foot, more marked on the left. The examination of both hips was normal.

**Figure 2:**
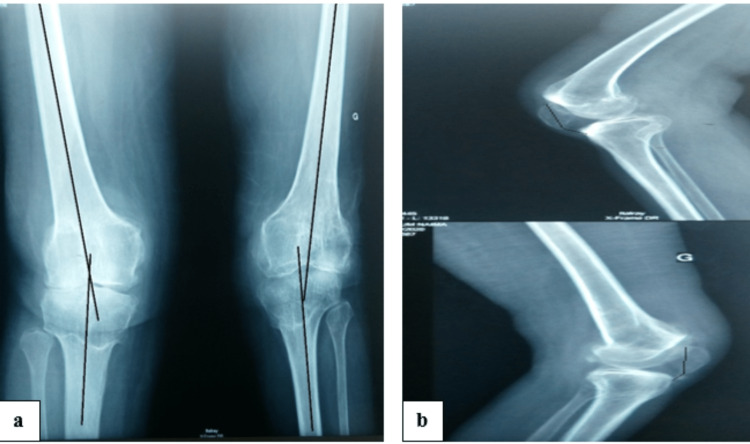
Frontal (a) and lateral (b) preoperative X-ray showing severe valgus knee with patella baja for both knees.

An X-ray of both knees revealed a bilateral tricompartmental gonarthrosis, with a bilateral valgus (15° for the left knee and 13° for the right knee) and patella baja for both knees (Figure 2). Considering the patient's bilateral disability, the advanced stage of osteoarthritis (stage 4 Ahlbäck) and the presence of bilateral anteroposterior and mediolateral instability, hinge-type TKA was indicated for both knees 24 months apart.

**Figure 3:**
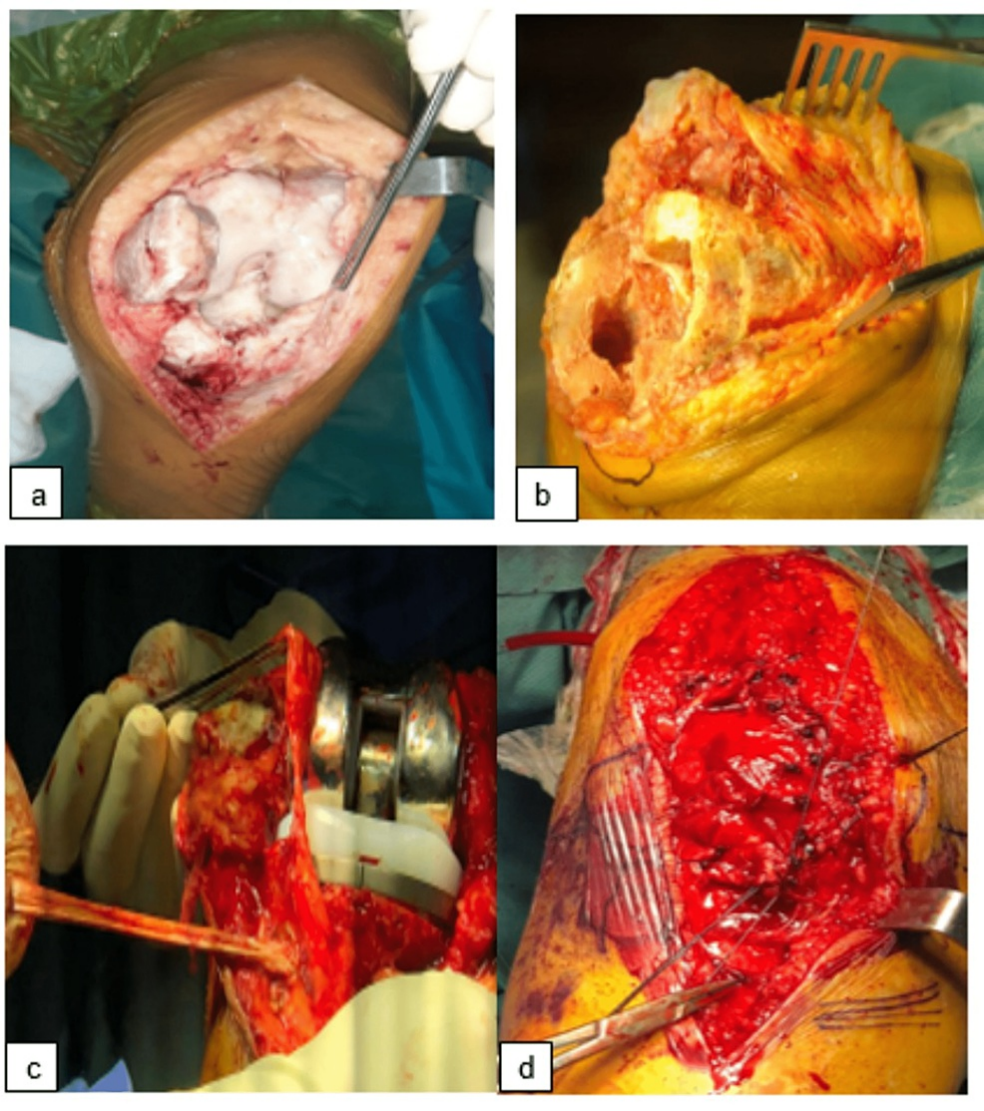
a) Intraoperative view showing the Z-plasty for the patellar tendon. b) Intraoperative view after femoral and tibial cuts with denervation of the patella. c,d) Intraoperative view showing the knee prosthesis and lengthening of the extensor apparatus by Z-plasty and reinforcement by a supra-patellar framing by the medial rectus.

The operation was performed with the patient in the supine position, with a tourniquet at the root of the thigh, a lateral callus for the operated knee, and a bolster for the feet. A prophylactic IV antibiotic dose was administered during the induction of anesthesia. After routine preparation and draping, an anterior incision with a transvastus approach was used. A patellar tendon Z-plasty was performed to dislocate the patella and correct the patella baja (Figure 3a). The anterior and posterior cruciate ligaments were absent from both knees after cleaning the joints - Hoffa's fat, meniscus and osteophyte, dislocation of the tibial plateau, and placement of intramedullary and extramedullary tibial cutting guides (2 mm cut of the plateau internal), and reaming of the tibial medullary canal with short rods then long rods with adapted reamers. After realization of distal femoral cuts, anterior, posterior, and anteroposterior chamfer with 5° of hip-knee-shaft angle (HKS), 0° of rotation for the two knees, we preserved a recurvatum of 9° for both knees, and then the realization of patellar denervation (Figure 3b). A test reduction was performed to ensure proper balance, stability, ROM, and patellar tracking. After having satisfactory stability, the corresponding final implants were selected after all trial implants were removed. Next, lengthening of the extensor apparatus by Z-plasty and reinforcement by a supra-patellar framing by the medial rectus were done (Figures 3c-3d]. Finally, careful washing with normal saline solution was performed before closure.

**Figure 4:**
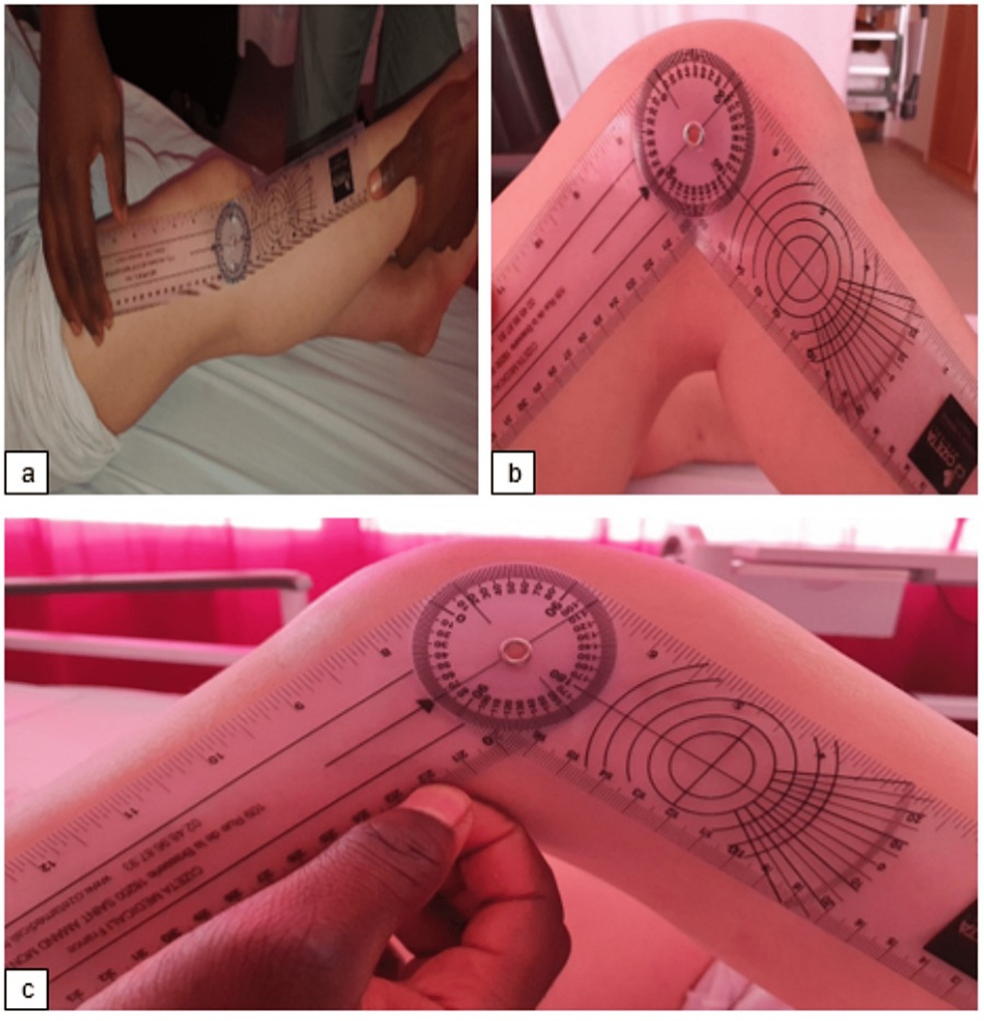
Functional results at the last follow-up. a) Patient with 9° of right knee recurvatum; b) patient with 85° of right knee flexion; and c) patient with 70° of left knee flexion.

**Figure 5:**
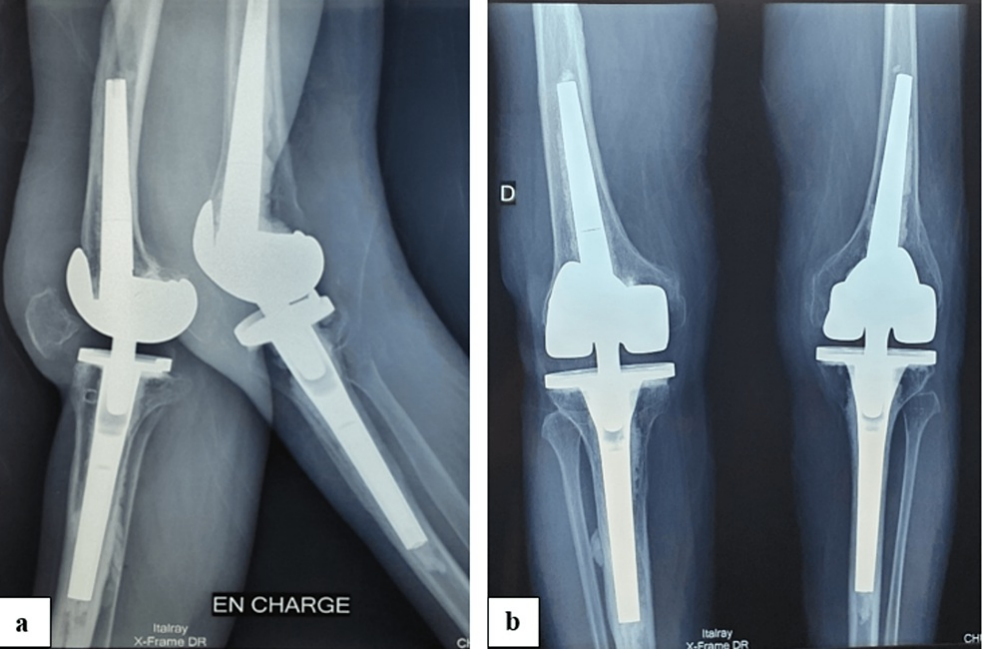
Lateral (a) and frontal (b) views of six months postoperative X-ray showing good alignment with no signs of loosening.

Postoperative rehabilitation occurred according to the standard protocol. At the last follow-up, the patient had no pain or signs of instability in either knee. The patient gained a range of motion of 9-90 after six months (Figure 4). The last follow-up X-rays showed good positioning of the TKA for both knees with no signs of prosthetic loosening (Figure 5 and Table 1).

**Table 1:** Preoperative and postoperative results for both knees. IKDC: The International Knee Documentation Committee Subjective Knee Form.

	Anatomic Valgus on Radiograph		Recurvatum/Flexion		Subjective IKDC score
Knee	Preoperative	Postoperative	Preoperative	Postoperative	
Right	13°	3°	40°/80	9°/85°	60
Left	15°	1°	40°/60°	9°/70°	65

## Discussion

People with a history of poliomyelitis are known to have degenerative joint disease. Many of them are now eligible for TKA as they are in the age range for the procedure [7]. However, only a few research studies have focused on TKA in poliomyelitis-affected limbs [3-5]. Overall, TKA in the poliomyelitis extremities offers sufficient pain relief but carries a significant risk of failure because of recurring instability and functional loss. Prasad A et al. conducted a systematic review, finding that although TKA of polio-affected limbs is a complicated procedure, it is beneficial in improving knee function and life quality in postoperative patients. Furthermore, in all studies included in this evaluation, functional outcomes and improvements reported by patient satisfaction were significant. After an average of six years, the overall revision rate was 7% [8]. Our case report shows promising results with TKA, such as reduced pain and increased knee stability, demonstrating the necessity of total knee replacement in polio survivors. According to research by Tigani D et al., patients with polio may benefit from a rotating hinged total knee replacement that allows hyperextension because it compensates for weak quadriceps muscles [7]. Jordan L et al., in their study including 15 patients, used eight posterior-stabilized TKA and eight constrained condylar knee (CCK) replacements. One case was treated with rotating hinged total knee replacement. The findings based on the American Knee Society (AKSS) score showed a rise in preoperative from 45 to 87 postoperatively. In all patients, the knee was stabilized, which included four patients with quadriceps strength below antigravity [5]. Similar results were obtained in the study by Tigani D et al., AKSS increased after surgery in all eight patients over a two-year follow-up [7].

The strength of the quadriceps muscle is a crucial predictor of knee function and hyperextension. The replacement procedure would be more challenging and complex if the quadricep muscle were weak during the preoperative physical assessment. A case of a bilateral total knee custom articulated prosthesis with a 30° recurvatum of the keel tibial in a poliomyelitis patient is described by Tardy N et al. The IKS knee score was better for both knees, with signs of radiological loosening on the left side. In fact, too much recurvatum can tear the posterior soft tissues and cause persistent pain. In this case, the rotating hinge prosthesis is designed to lessen and avoid this posterior mechanical stress. Therefore, the recurvatum of the knee must be maintained up to 10 degrees [9]. Rahman J et al. reported encouraging results using a personalized prosthesis Stanmore Modular Individualised Lower Extremity System (SMILES) for 13 patients, with five degrees of built-in hyperextension, femoral and tibial stem length. Diameter, curvature, and angulation are customized based on preoperative biplanar X-ray imaging to optimize fixation and compensate for deformities and bone loss [1]. In our case, the quadriceps strength was 3/5, which allowed to preserve 9° of recurvatum.

## Conclusions

Due to bone and soft tissue defects, total knee replacement is a challenging surgery for poliomyelitis patients. Therefore, we propose that patients with polio-affected limbs who have a severely unstable knee, with or without significant loss of quadriceps power, should have a rotating hinge total knee prosthesis. To ascertain if these positive results are maintained, longer follow-up is necessary.
